# Thermoreversible Hydrocolloid Blends for Structurally Stable Reheated Carrot Purée in Dysphagia Management

**DOI:** 10.3390/foods14132248

**Published:** 2025-06-25

**Authors:** Narmatha Maran, Jorry Dharmawan, Kelvin K. T. Goh

**Affiliations:** 1 Singapore Institute of Technology, Food, Chemical and Biotechnology Cluster, 1 Punggol Coast Road, Singapore 828608, Singapore; narmatha.m.nm@gmail.com (N.M.); jorry.dharmawan@singaporetech.edu.sg (J.D.); 2 School of Food Technology and Natural Sciences, Massey University, Private Bag 11 222, Palmerston North 4442, New Zealand

**Keywords:** dysphagia, texture-modified foods, IDDSI, methylcellulose, heat-induced gelation, syneresis, shape retention, moulded foods, purées, soft foods

## Abstract

Oropharyngeal dysphagia is a common condition among older adults and individuals with neurological disorders, necessitating the use of texture-modified foods (TMFs) to ensure safe swallowing; however, reheating often leads to syneresis and structural breakdown, compromising both functionality and patient acceptability. This study aimed to evaluate the efficacy of single and binary hydrocolloid systems for improving the thermal and structural stability of moulded carrot purée formulated to meet International Dysphagia Diet Standardisation Initiative (IDDSI) Level 4 standards. The main methods involved preparing purées with various hydrocolloid combinations, assessing gel strength, shape retention, and syneresis following steaming, and validating results using commercial moulds. Thermoreversible methylcellulose (Benecel™ A4M) was the most effective single-component system, while binary blends of A4M with locust bean gum (LBG)—specifically B2 (1.5% A4M + 0.5% LBG) and B3 (1.5% A4M + 1% LBG)—demonstrated superior structural integrity, with height retention of 80 ± 2% (B2) and 85 ± 2% (B3), and reduced syneresis (~22 ± 1% and ~19 ± 3%, respectively; *p* < 0.05), both meeting IDDSI requirements. In contrast, formulations containing agar, xanthan, or carboxymethylcellulose exhibited poorer shape fidelity, likely due to matrix-disrupting interactions. These findings indicate that A4M-LBG blends offer a practical solution for producing reheatable, visually recognisable meals for individuals with moderate-to-severe dysphagia.

## 1. Introduction

Oropharyngeal dysphagia (OD) is a swallowing disorder linked to serious health risks, including malnutrition, aspiration pneumonia, and increased mortality [1,2]. These risks are particularly pronounced in older adults, where OD is not only more common but also increasingly recognised as a geriatric syndrome [3,4]. While prevalence in the general population is estimated to range from 2.3% to 16% [5], rates are significantly higher in clinical settings. A recent systematic review found OD to be present in 36.5% of hospitalised patients (95% CI: 29.9–43.6), 42.5% in rehabilitation facilities (95% CI: 35.8–49.5), and 50.2% in nursing homes (95% CI: 33.3–67.2) [6]. Given its far-reaching consequences, oropharyngeal dysphagia (OD) places a significant burden on healthcare systems worldwide. Effective management depends on coordinated input from multidisciplinary teams—including speech-language pathologists, gastroenterologists, otolaryngologists, dietitians, and food scientists—who work collectively to reduce the risks of aspiration, malnutrition, and related complications. At the same time, they play a critical role in ensuring that meals remain nutritionally adequate, safe to swallow, and sensorially acceptable. This is particularly important in the context of institutional care, where food consistency must comply with standardised safety guidelines while meeting the individual dietary needs of patients. By supporting the development and delivery of tailored meal solutions—especially those that preserve the sensory and visual appeal of conventional foods—these professionals contribute meaningfully to improved clinical outcomes and a better quality of life for individuals living with dysphagia.

Texture-modified foods (TMFs) and thickened fluids are widely used in dysphagia management to promote safer and more efficient swallowing. These modifications reduce the risk of aspiration and allow better control over bolus flow and intake [7,8]. TMFs are mechanically and rheologically tailored to achieve a soft, cohesive texture with adequate lubrication, supporting safe oral processing and smooth transit through the pharynx [9,10]. However, despite their functional role, TMFs often lack sensory appeal, leading to reduced intake, heightened risk of malnutrition, and increased food wastage—particularly in aged-care facilities and hospital settings [11,12]. A major limitation of TMFs, beyond their complex mechanical and rheological attributes, is their often unappetising visual appearance, which markedly diverges from that of conventional meals. A survey by Smith, et al. [13] revealed that dysphagia patients described these meals as resembling “wet dog food”, reflecting dissatisfaction with their texture and visual presentation. Such negative perceptions may diminish mealtime enjoyment and contribute to social withdrawal or self-consciousness [14]. Food shaping through moulding or 3D printing offer a promising strategy to address these concerns. By replicating the form of conventional foods, shaped purées can improve recognisability and enhance visual appeal. Recent evidence suggests that these approaches increase overall liking and engagement during mealtime [15], potentially leading to better compliance and nutritional outcomes.

Hydrocolloids, including starches and non-starch polysaccharides, are commonly incorporated into TMF formulations to improve viscosity, texture, and structural stability [16,17]. These properties are particularly important for reshaped purées, which must retain their form and texture during storage and reheating. However, thermal processing often introduces challenges such as syneresis, surface collapse, and textural degradation, all of which can compromise both safety and palatability [18,19]. While hydrocolloids are typically used to address these issues, their effectiveness in maintaining shape fidelity after reheating remains underexplored.

Among the available commercial hydrocolloids, hydroxypropyl methylcellulose (HPMC) and methylcellulose (MC) are of particular interest due to their unique thermoreversible gelation properties. Unlike most gelling agents that solidify upon cooling, HPMC and MC form stable gels when heated, through mechanisms involving coil-to-helix transitions and hydrophobic interactions [20]. Despite this unique functionality, their application in dysphagia-specific foods—particularly for enhancing reheating stability and shape retention in moulded purées—has not been systematically investigated. This presents a significant opportunity to improve the design of reheatable, visually structured meals tailored to the International Dysphagia Diet Standardisation Initiative (IDDSI) framework.

This study investigates the potential of selected hydrocolloids—methylcellulose (MC) and hydroxypropyl methylcellulose (HPMC)—to improve the structural stability of moulded carrot purée subjected to steaming. Carrot purée was selected as a model system due to its favourable nutritional profile, consistent softening behaviour upon cooking, and well-characterised rheological properties, which make it ideal for evaluating texture-modifying strategies in dysphagia-oriented foods. These attributes also facilitate the systematic assessment of hydrocolloid interactions in maintaining textural integrity and shape retention under thermal stress. The study further explores the combined effects of thermoreversible hydrocolloids with agar, locust bean gum (LBG), xanthan gum, and carboxymethylcellulose (CMC), focusing on textural behaviour before and after reheating, with a particular emphasis on compliance with the IDDSI Level 4 criteria [21] to ensure safety and suitability for individuals with moderate-to-severe dysphagia.

## 2. Materials and Methods

### 2.1. Materials

Methylcellulose (MC) samples of varying molecular weights—Benecel™ A4C (150,000 g/mol), A4M (300,000 g/mol), MX (500,000 g/mol), and MX100 (700,000 g/mol)—were kindly provided by Ashland Singapore Pte Ltd. (Singapore). These were manufactured by Ashland Industries Belgium BV, Zwijndrecht, Belgium. Hydroxypropyl methylcellulose (HPMC; Benecel™ E4M, 300,000 g/mol) and carboxymethylcellulose sodium salt (CMC; Blanose™ CMC 7H9FDN) were also obtained from Ashland Singapore Pte Ltd. (Singapore), with HPMC manufactured by Ashland Industries Belgium BV, Zwijndrecht, Belgium and CMC manufactured by Ashland France S.A.S., Levallois-Perret, France. Agar (Agar Agar GS900) was sourced from Natural Colloids Industries Pte Ltd. (Singapore), with raw materials imported from Brazil. Xanthan gum (Keltrol^®^ AP) was supplied by CP Kelco Singapore Pte Ltd. (Singapore), produced produced by CP Kelco (China) Co., Ltd., Wuxi, China. Locust bean gum (LBG RL200) was also supplied by CP Kelco Singapore Pte Ltd. (Singapore), manufactured by CP Kelco ApS, Lille Skensved, Denmark. Fresh carrots (*Daucus carota*) were purchased locally from FairPrice Supermarket, Singapore.

### 2.2. Temperature Sweep Analysis of Methylcellulose and Hydroxypropyl Methylcellulose

To investigate the thermal gelation behaviour of cellulose ether hydrocolloids, a temperature sweep test was conducted using four types of methylcellulose (MC; Benecel™ A4C, A4M, MX, and MX100) and one hydroxypropyl methylcellulose (HPMC; Benecel™ E4M), each prepared at 1% *w*/*w* in distilled water. Hydrocolloid powders were dispersed and hydrated by continuous mixing at 600 rpm and 25 °C for 1 h on a magnetic hot plate stirrer to ensure complete dissolution.

Rheological measurements were carried out using an Anton Paar MCR 302 rheometer (Anton Paar GmbH, Graz, Austria) equipped with a concentric cylinder geometry (CC27). Temperature-dependent viscoelastic properties were assessed by performing a controlled temperature sweep from 25.0 ± 0.1 °C to 90.0 ± 0.01 °C, followed by a reverse sweep back to 25.0 ± 0.1 °C. The test was conducted at a constant frequency of 1 Hz and strain amplitude of 0.1%, within the linear viscoelastic region (LVR). The heating and cooling rate was maintained at 2 °C/min to simulate gradual thermal transitions relevant to food processing. The aim of this analysis was to determine the sol–gel and gel–sol transition temperatures, as well as the corresponding changes in storage modulus (G′) and loss modulus (G″), to characterise the thermoreversible or irreversible gelation behaviour of each hydrocolloid. Data acquisition was managed via RheoCompass™ software (version V1.33.615), and subsequent analysis was performed using Microsoft Excel. All measurements were conducted in duplicates.

### 2.3. Preparation of Carrot Purée and the Addition of Hydrocolloids (MC and HPMC)

Carrot purée was prepared by adapting the procedure outlined by Peh et al. [22], which involved peeling, dicing, steaming until tender, and blending with potable water to achieve a smooth consistency suitable for dysphagia management. Specifically, fresh carrots were thoroughly washed, peeled, and chopped before being boiled in water for 20 min. The softened carrots were then transferred to a Waring blender (Model MX1500XTXP, Waring Commercial, Torrington, CT, USA) and blended until a smooth, homogeneous purée was obtained. A total of 250 g aliquot of the purée was subsequently heated in a Thermomix TM6 (Vorwerk, Wuppertal, Germany) at 80 °C for 10 min using the mixing blade. During this step, methylcellulose (MC; Benecel™ A4C, A4M, MX, MX100) or hydroxypropyl methylcellulose (HPMC; Benecel™ E4M) was incorporated at varying concentrations (0.5%, 1.0%, and 1.5% *w*/*w*) to ensure uniform dispersion and adequate hydration. Hydrocolloids were added directly to the purée under continuous mixing.

Each formulation consisted of 75% (*w*/*w*) carrot purée and 25% (*w*/*w*) water, adjusted to account for the specific hydrocolloid concentration. A total of 16 formulations—including a control (0% hydrocolloid)—were prepared to assess the independent effects of MC and HPMC on structural integrity and syneresis.

The purée mixtures were cast into cylindrical silicone moulds (dimensions: 39.6 × 19.3 × 5.1 cm; Silikomart, Venice, Italy) and frozen at –18 °C for 24 h. Prior to analysis, samples were demoulded and steamed in a UNOX combi oven (Model XEVC-0511-EPRM, UNOX S.p.A., Cadoneghe, Italy)at 100 °C for 20 min under 100% relative humidity and maximum fan speed, to evaluate shape retention and thermal syneresis.

### 2.4. Evaluation of Carrot Purée Functionality with Single and Combined Hydrocolloids

Following preliminary screening, methylcellulose A4M (MC-A4M) was selected for further investigation due to its favourable performance. It was subsequently assessed in combination with other hydrocolloids—namely agar, locust bean gum (LBG), xanthan gum and carboxymethylcellulose (CMC)—to optimise the textural and structural properties of carrot purée. The formulations, detailed in Table 1, were prepared by dispersing the designated hydrocolloids into a standardised carrot purée–water matrix. Blending was conducted using a Thermomix TM6 (Vorwerk, Germany), comprising two mixing phases: 10 min at 25 °C, followed by 10 min at 80 °C to ensure adequate hydration and uniform dispersion.

Each formulation was cast into cylindrical silicone moulds (39.6 × 19.3 × 5.1 cm) and frozen at −18 °C for 24 h. To assess shape retention and control of syneresis under thermal stress, frozen samples were demoulded and steamed at 100 °C for 20 min, as described previously. Steaming at 100 °C under humid conditions simulates reheating protocols in commercial kitchens and healthcare food services, ensuring relevance to practical application. Post-steaming, visual observations were recorded, the height of each sample measured at the centre, and the amount of liquid released due to syneresis determined by weighing the expelled fluid. All measurements were conducted in triplicate.

### 2.5. Evaluation of Carrot Purée Texture Against IDDSI Level 4 Standards

The texture of the carrot purée was evaluated for compliance with the International Dysphagia Diet Standardisation Initiative (IDDSI) Level 4 (Puréed) criteria [21] using the Fork Pressure Test and the Spoon Tilt Test. These assessments were carried out both immediately after blending (~25 °C) and following the steaming process (~70 to 80 °C) to verify consistency of texture throughout processing.

Texture was evaluated at ~25 °C (post-blend) and again at ~70–80 °C (post-steam) meeting the following criteria: (i) no lumps observed; (ii) for the fork-drip test, the sample sat in a mound and did not drip through the fork; and (iii) for the spoon tilt test, the sample held its shape on the spoon, slid off cleanly when tilted (not sticky), and slumped slowly on a flat plate, in accordance with the 2020 IDDSI audit tool for Level 4 [21,23]. These quantitative thresholds confirm that product flow, cohesion, and consistency remain within Level 4 specifications throughout processing.

### 2.6. Statistical Analysis

Quantitative analysis of structural integrity (measured by height retention) and syneresis was conducted using Minitab Statistical Software (Version 21, Minitab LLC, USA). A one-way analysis of variance (ANOVA) was employed to determine statistically significant differences among the formulations. Where significant main effects were identified, Tukey’s post hoc test was applied for pairwise comparisons. Alphabetic groupings were assigned to the mean values to indicate statistically distinct groups across treatments. Significance was established at *p* < 0.05. Results are reported as mean ± standard deviation (SD) based on triplicate measurements, with error bars representing the SD in graphical presentations.

## 3. Results and Discussion

### 3.1. Thermal Gelation and Reversibility of Cellulose Ether Hydrocolloids

**Figure 1:**
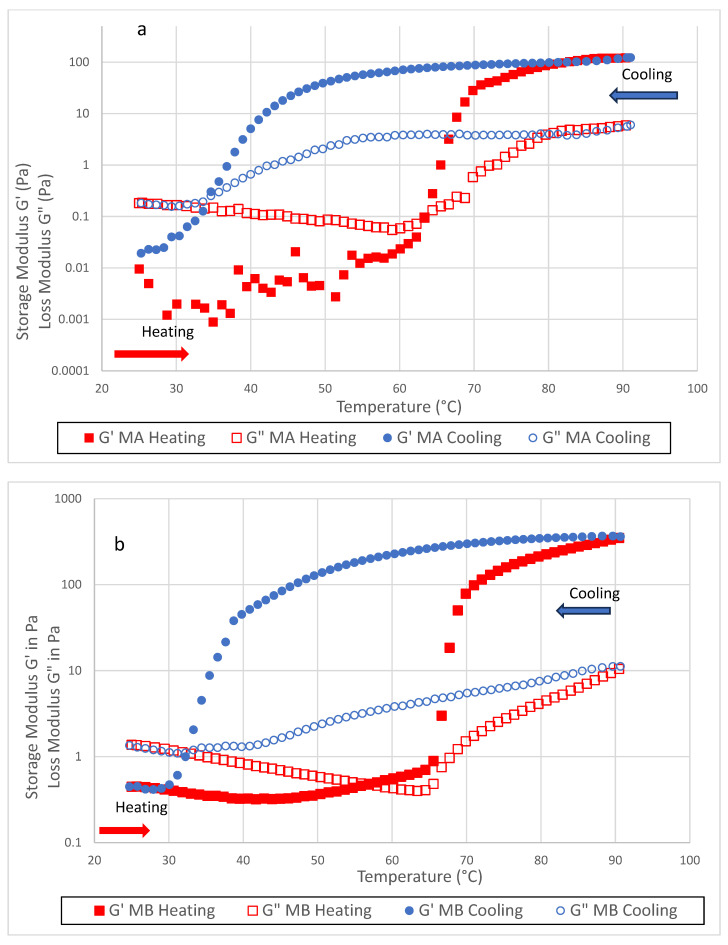

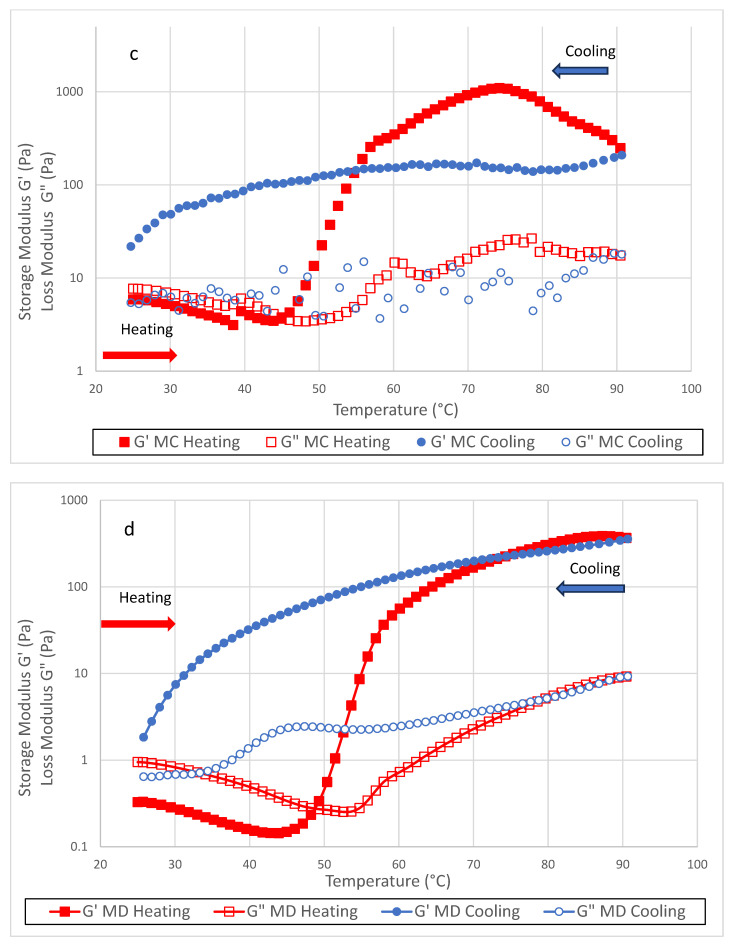

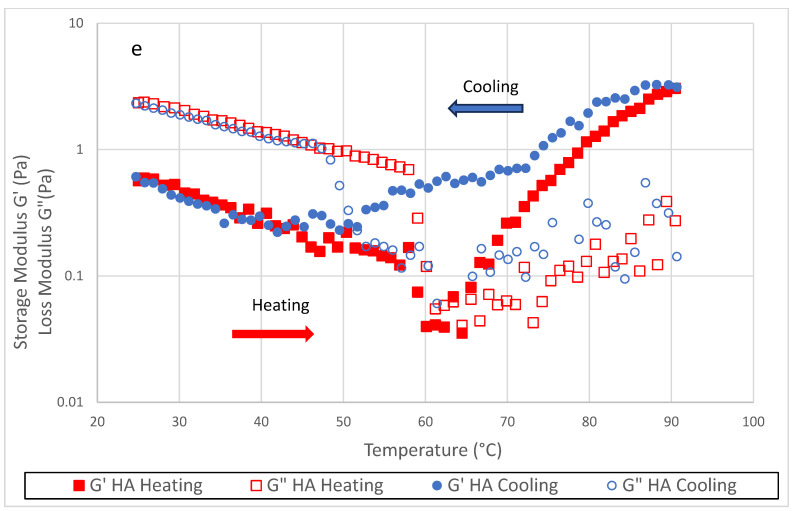
Temperature sweep analysis from 25.0 °C to 90.0 °C (heating, red symbols) and 90.0 °C to 25.0 °C (cooling, blue symbols) for (**a**) MA (Benecel™ A4C), (**b**) MB (Benecel™ A4M), (**c**) MC (Benecel™ MX), (**d**) MD (Benecel™ MX100), and (**e**) HA (HPMC, Benecel™ E4M) Storage modulus (G′, closed symbols) and loss modulus (G″, open symbols) are plotted as functions of temperature.

Temperature sweep analysis revealed two distinct thermal behaviours among the cellulose ether hydrocolloids tested (Figure 1): thermoreversible gelation in A4C (MA), A4M (MB), and E4M (HA), and irreversible gelation in MX (MC) and MX100 (MD). The sol–gel and gel–sol transition temperatures, as well as G′ values at 90 °C, are presented in Table 2. These transitions are critical for ensuring that the purée remains stable and retains its moulded shape at serving temperatures in aged-care and hospital settings.

Samples MA, MB, and HA followed a gelation mechanism characteristic of cellulose ethers, involving thermal dehydration and subsequent hydrophobic association. At ambient temperatures, polymer chains remain solvated through hydrogen bonding with water molecules. As the temperature increased, hydrogen bonds between hydroxyl groups and water molecules were disrupted, exposing hydrophobic methyl or hydroxypropyl groups. These groups promoted intermolecular association and network formation through hydrophobic interactions. During cooling, rehydration of the polymer chains weakened hydrophobic interactions, resulting in disassembly of the gel structure and a transition back to the sol state [20,24]. This dynamic transition is strongly influenced by molecular weight, chain mobility, and the degree and pattern of substitution—namely, the availability of methyl, carboxyl, and hydroxyl groups—which modulate intermolecular hydrogen bonding and hydrophobic associations [25].

As shown in Table 2, MA (A4C) and MB (A4M) gelled at ~62 °C and ~57 °C, respectively, and returned to sol at ~33 °C and ~32 °C. Their respective G′ values at 90 °C were ~122 Pa and ~349 Pa. However, closer examination of modulus decay during cooling reveals critical differences. For MA (A4C), G′ decreased gradually from ~122 Pa at 90 °C to ~39 Pa at 50 °C. Between 50 °C and the gel–sol transition (~33 °C), G′ declined sharply to ~0.2 Pa, indicating a rapid collapse of the gel network near the crossover temperature (Figure 1a). In contrast, MB (A4M) exhibited a more gradual reduction in G′, declining from ~349 Pa at 90 °C to ~138 Pa at 50 °C before steeply dropping to ~1.1 Pa at ~32 °C (Figure 1b). These results demonstrate that both systems retained substantial structural integrity between 90 °C and 50 °C—a range encompassing the typical serving temperatures of warm meals (55–65 °C) in healthcare and aged-care environments [10]. This stability within intermediate temperature ranges indicates that thermoreversible hydrogels resist rapid structural breakdown, maintaining sufficient gel integrity throughout plating, transportation, and initial consumption phases in dysphagia care settings.

For applications requiring prolonged holding or reheating, such as institutional service settings, the eventual loss of network integrity below ~40 °C must still be considered in formulation design. The performance of these systems will depend on their stability under real-time temperature decline and mechanical stress. For HA (E4M), thermoreversible gelation was also observed, with a sol–gel transition at ~63 °C and a gel–sol transition at ~51 °C. Nonetheless, its gel strength was considerably lower than MA and MB, with G′ reaching only ~91 Pa at 90 °C (Figure 1e). The low modulus throughout the heating and cooling cycles indicates a weaker gel network, which may be attributed to the presence of hydroxypropyl groups. These bulky substituents introduce steric hindrance that limits efficient chain entanglement and hydrophobic association during gel formation, ultimately reducing structural cohesion [26]. As a result, E4M may be less suited for formulations where moderate-to-high mechanical strength is required for reheating or moulded presentation.

In contrast, the methylcellulose variants MX (MC) and MX100 (MD) exhibited irreversible gelation, characterised by no gel–sol transition and the absence of a G′–G″ crossover upon cooling. These systems formed strong, thermally stable gels that retained their structure throughout the entire temperature cycle. MC (MX) exhibited gelation onset at ~45 °C and reached a G′ of ~208 Pa at 90 °C. During cooling, G′ decreased gradually to ~126 Pa at 50 °C but remained well above G″, indicating retention of the gel structure (Figure 1c). Similarly, MD (MX100) gelled at ~49 °C with a peak G′ of ~367 Pa at 90 °C, declining to ~76 Pa at 50 °C. Again, no G′–G″ crossover was observed, confirming the stability of the gelled network (Figure 1d).

Although both systems experienced some modulus decline with decreasing temperature, the absence of network disruption is indicative of densely entangled polymer matrices reinforced by strong hydrophobic associations. A higher degree of substitution supplies more hydrophobic sites along the backbone, creating additional junction zones and further stabilising the gel. This one-way (irreversible) gelation is therefore driven by the combined effects of high molecular weight, limited chain mobility, and an adequate degree of substitution, all of which prevent rehydration and collapse during cooling [20,24,25].

These irreversible gels are thus well suited to applications requiring persistent mechanical strength and thermal stability, such as steam-regenerated or held-at-temperature meals, where deformation or collapse must be avoided. Their robust mechanical performance across processing and serving conditions makes them promising for moulded purées, 3D-printed shapes, and other visually structured TMFs intended for individuals with moderate-to-severe dysphagia.

### 3.2. Effect of Methylcellulose and Hydroxypropyl Methylcellulose on the Stability of Carrot Purée

**Figure 2:**
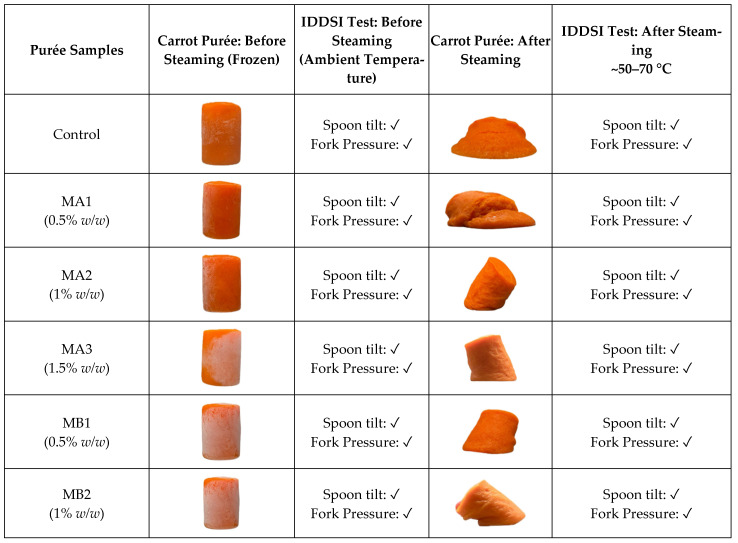

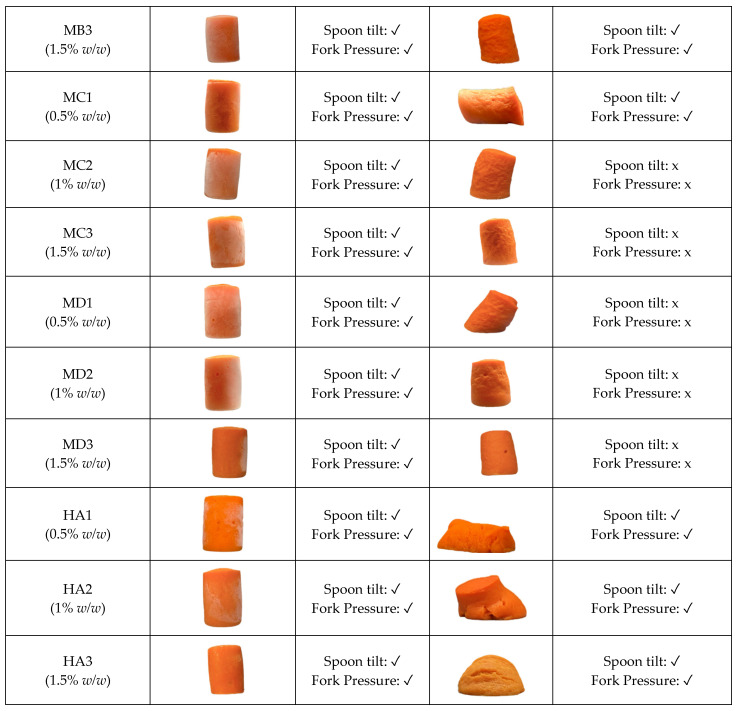
Carrot purée samples before and after steaming, evaluated using the IDDSI spoon tilt test and fork pressure test (Level 4). Samples were formulated with Benecel™ A4C, A4M, MX, and MX100, as well as hydroxypropyl methylcellulose (HPMC, Benecel™ E4M), denoted as MA, MB, MC, and MD, each at 0.5%, 1%, and 1.5% *w*/*w*. The ✓ and ✗ symbols indicate whether the sample passes or fails the IDDSI Level 4 test.

The performance of four methylcellulose (MC) variants and one hydroxypropyl methylcellulose (HPMC) variant was evaluated to identify the most effective hydrocolloid for maintaining the structural integrity of carrot purée under thermal processing, while ensuring compliance with IDDSI Level 4 standards. Visual assessments before and after steaming (Figure 2), combined with IDDSI characterisation, provided insights into the role of hydrocolloids in structure retention and safe swallowability. The percentage of drip loss due to syneresis was also measured.

Prior to steaming, all formulations exhibited a well-defined cylindrical shape (sample height 4.5 cm), achieved by freezing the samples followed by removal from the mould. However, steaming—a moist heat method involving both conduction and convection—significantly altered the shape of the purée samples. The control sample, devoid of hydrocolloids, completely lost its shape upon steaming, although it fulfilled IDDSI Level 4 requirements.

**Figure 3:**
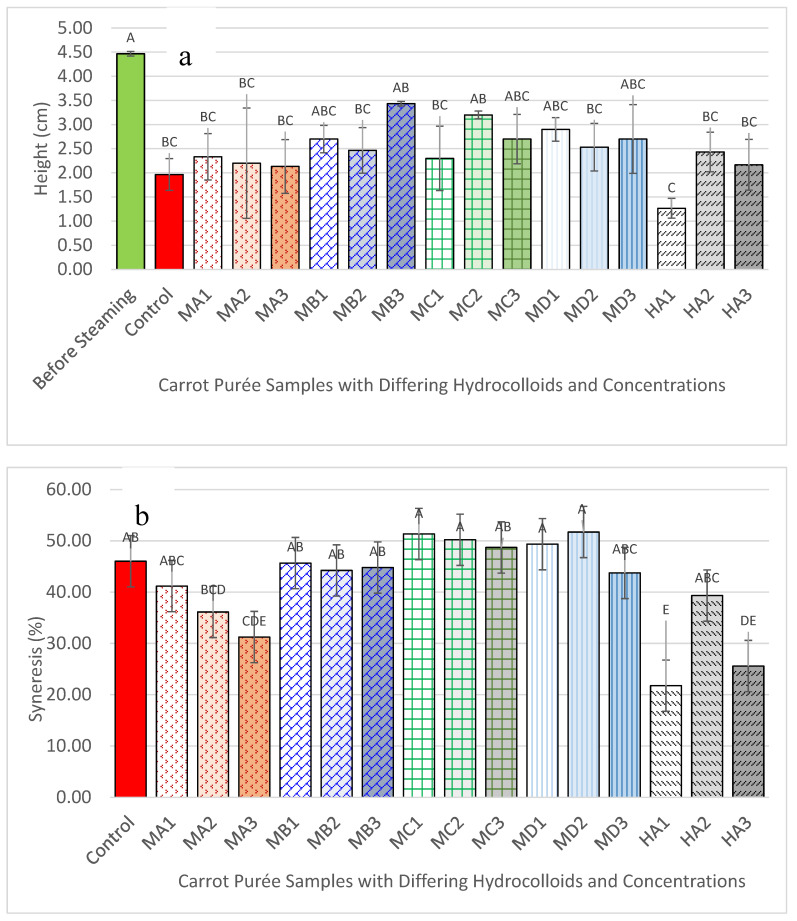
Bar graphs showing (**a**) the mean height and (**b**) % syneresis of moulded carrot purée samples during steaming. Hydrocolloid treatments are denoted as follows: Control (carrot purée without hydrocolloids); MA (Benecel™ A4C) at 0.5%, 1.0%, and 1.5% (MA1, MA2, MA3); MB (Benecel™ A4M) at 0.5%, 1.0%, and 1.5% (MB1, MB2, MB3); MC (Benecel™ MX) at 0.5%, 1.0%, and 1.5% (MC1, MC2, MC3); MD (Benecel™ MX100) at 0.5%, 1.0%, and 1.5% (MD1, MD2, MD3); and HA (Benecel™ E4M, HPMC) at 0.5%, 1.0%, and 1.5% (HA1, HA2, HA3). Error bars represent the mean ± standard deviation (SD) from three replicates (*n* = 3). Different uppercase letters denote statistically significant differences between samples (*p* < 0.05).

Purée samples containing hydrocolloids, except for MA1 and all HA samples, partially retained the shape of the mould. Notably, only MB3 (Benecel™ A4M at 1.5%) maintained its shape best among the samples after steaming and passed the IDDSI Level 4 criteria, indicating that it had formed the most suitable gel network for the carrot purée system. Despite this, MB3 still exhibited a loss of approximately 24% in height compared to its pre-steamed state, although this reduction was not statistically significant (*p* < 0.05) (Figure 3a).

Despite its superior structure, MB3 exhibited pronounced syneresis (Figure 3b), suggesting poor water-binding capacity. This high syneresis explains the loss in height. Excessive fluid separation is undesirable in dysphagia-friendly foods due to its impact on appearance and potential risks for aspiration [27]. While MC2, MC3, MD2, and MD3 retained their shapes, they were deemed inappropriate for dysphagia consumption due to an overly firm, stress-ball-like texture that did not meet IDDSI Level 4 pressure fork and spoon tilt test thresholds.

Conversely, samples such as MA1–3, MB1–2, MC1, and HA1–3, though lacking structural integrity after steaming, successfully passed IDDSI Level 4 testing. This illustrates a critical insight: passing IDDSI does not necessitate shape retention, and some formulations may still be suitable despite poor mould fidelity.

Syneresis showed a non-monotonic response across the HPMC series (Figure 3b). The most dilute gel, HA1 (0.5%), and the most concentrated gel, HA3 (1.5%), retained water equally well, whereas the mid-range sample, HA2 (1.0%), released significantly more liquid (*p* < 0.05). This pattern can be rationalised by each formulation’s position relative to the chain-overlap concentration (c*). HA1 lies just above c*, so the chains begin to entangle and trap water. At 1.0% (HA2) additional hydroxypropyl groups bind more water and raise viscosity—a well-documented effect of HPMC [26] —but the network is still below the point at which the chains form a continuous, percolated mesh; on cooling, some of this loosely bound water drains out. By 1.5% (HA3), the chain density is high enough to create a fully connected network, and the extra junction zones offset the hydrophilic pull of the substituents, keeping the water in place. These results indicate that minimising syneresis is not simply a matter of adding more HPMC: polymer concentration must exceed the entanglement threshold, and the degree of hydrophilic substitution must be balanced with network connectivity. These findings highlight the trade-offs between mechanical robustness, water-holding capacity, and IDDSI compliance, and form the basis for subsequent optimisation through hydrocolloid combinations.

### 3.3. Optimisation of Carrot Purée Stability Using Hydrocolloid Combinations

**Figure 4:**
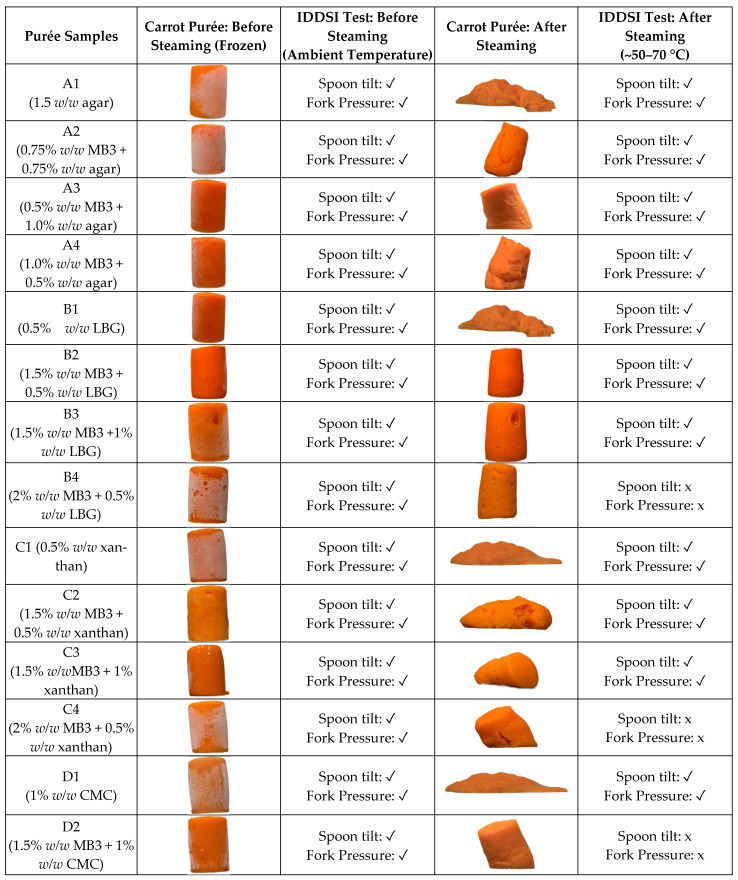
Visual assessment of carrot purée samples before and after steaming, evaluated using IDDSI Level 4 criteria (spoon tilt and fork pressure tests). Samples were formulated with Benecel™ A4M (1.5%) in combination with agar, locust bean gum (LBG), xanthan gum, or carboxymethylcellulose (CMC), at concentrations ranging from 0.5% to 1.5% (*w*/*w*). The ✓ and ✗ symbols indicate whether the sample passes or fails the IDDSI Level 4 test.

**Figure 5:**
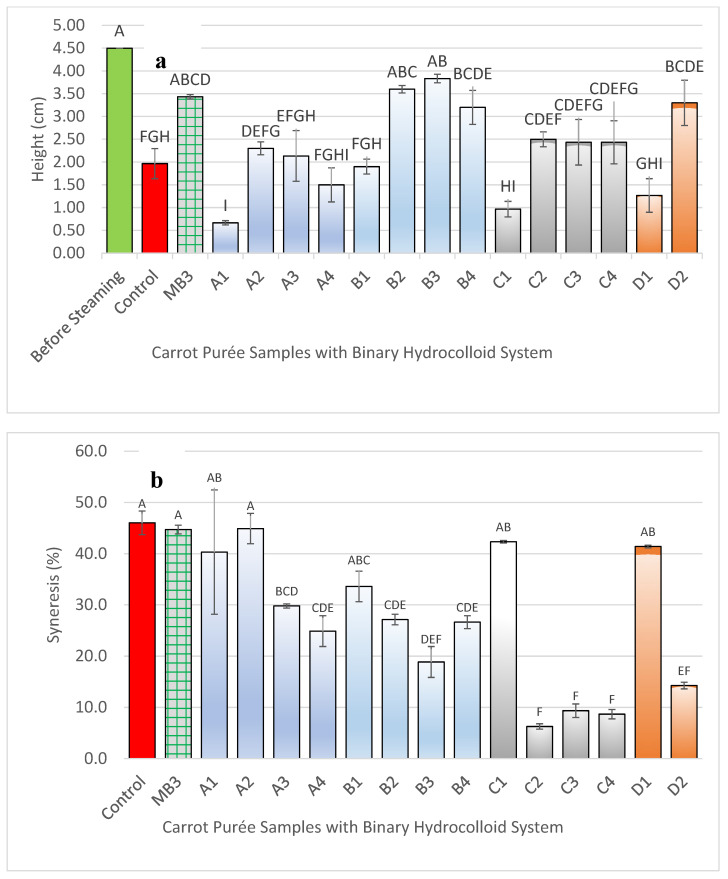
Bar graphs showing (**a**) mean height retention and (**b**) percentage syneresis of carrot purée samples after steaming. Formulations include: MB3 (Benecel™ A4M 1.5%), A1–A4 (agar-containing systems), B1–B4 (LBG-containing systems), C1–C4 (xanthan-containing systems), and D1–D2 (CMC-containing systems). Error bars represent mean ± SD of three replicates. Different letters indicate statistically significant differences among samples (*p* < 0.05).

Building on the hydrocolloid screening results, Benecel™ A4M at 1.5% (MB3) was identified as the most effective single-hydrocolloid formulation for height retention. However, MB3 alone exhibited substantial syneresis after steaming and borderline compliance with IDDSI Level 4 criteria. To address the limitations of single-component systems, binary hydrocolloid formulations were developed by combining Benecel™ A4M (1.5%) with various commercial polysaccharides—including agar, locust bean gum (LBG), xanthan gum, and carboxymethylcellulose (CMC)—at concentrations ranging from 0.5% to 1.5% (*w*/*w*). These combinations were designed to enhance network strength, reduce water migration, and meet the textural requirements for dysphagia-friendly applications. Figure 4 presents a visual assessment of the carrot purée samples before and after steaming, evaluated using IDDSI Level 4 spoon tilt and fork pressure tests. Corresponding quantitative data on height retention and percentage syneresis are shown in Figure 5. Differences among samples were statistically significant (*p* < 0.05), as indicated by distinct letter groupings.

B2 and B3 Systems: A4M (1.5%) + LBG (0.5% or 1%): 

Among all the combinations, B2 (A4M 1.5% + LBG 0.5%) and B3 (A4M 1.5% + LBG 1%) exhibited superior performance in terms of shape retention and IDDSI Level 4 compliance. Both samples passed the fork pressure and spoon tilt tests and showed significantly reduced syneresis compared to MB3 alone (~22 ± 1% for B2; ~19 ± 3% for B3 vs. ~45 ± 0.9% for MB3; *p* < 0.05), with corresponding height retention of 80 ± 2% (B2), 85 ± 2% (B3), and 76 ± 1% for MB3.

Although these gravimetric syneresis values (19–22%) appear substantial, they were obtained in tall silicon moulds (~45 mm) that accentuate drainage. When the same formulations were portioned in the shallower (~20 mm) service trays used for dysphagia meals (see Section 3.5), there was negligible visible syneresis, thereby meeting the IDDSI Level 4 requirement that “no thin liquid should separate from the purée” [21]. Quantitative syneresis data for texture-modified purées are scarce: a recent study on retorted rice–fish meals for elderly diners shows that syneresis testing, alongside Level-4 utensil checks, is now treated as an essential quality metric [28], while a contemporary hydrocolloid review notes that most papers still report syneresis only qualitatively and calls for standardised benchmarks [29]. One of the few available studies found that enriching a commercial peach purée with high-amylose retrograded starch reduced liquid loss from ~69% to ~23% while still passing Level 4 utensil tests [30]. Set against these limited benchmarks—and given the high height retention (80–85%) and full utensil-test compliance observed—B2 and B3 appear promising. These improvements are attributed to the complementary structuring mechanisms of A4M and LBG. Nevertheless, future work should characterise the rheology of the expressed serum and include clinical swallowing assessments to confirm safety and acceptability.

A4M forms thermoreversible gels through hydrophobic association upon heating, providing a backbone for matrix formation. LBG, a galactomannan, enhances this network by contributing hydrogen bonding and molecular entanglement, particularly under freeze–thaw conditions. This synergy results in a cohesive, elastic gel matrix with enhanced water-holding capacity and structural resilience. The higher LBG level in B3 led to denser molecular interactions and a more pronounced capillary barrier to water migration, resulting in marginally improved height retention relative to B2.

These findings are consistent with previous studies. Xu, et al. [31] demonstrated that LBG enhances starch gel stability by increasing water-binding capacity and reinforcing the gel network. Similarly, Muadklay and Charoenrein [32] reported marked reductions in syneresis in tapioca systems with the addition of small amounts of LBG. In this study, the incorporation of LBG alongside methylcellulose (A4M 1.5%) appears to mitigate excessive chain association and to reduce the negative effects seen when A4M is used at higher concentrations, leading to a more stable and cohesive gel that holds water more effectively.

B4 System: A4M (2%) + LBG (0.5%):

While the B4 formulation demonstrated strong shape retention and minimal syneresis, it did not comply with IDDSI Level 4 requirements due to excessive firmness. The higher concentration of A4M likely produced an over-structured gel that lacked sufficient compressibility under fork pressure, making it unsuitable for individuals with dysphagia. This underscores the need to balance gel strength with ease of oral processing to ensure both safety and compliance with IDDSI criteria.

B1 (LBG 0.5% alone):

In contrast, the B1 sample was unable to retain its structure after steaming, despite showing reduced syneresis. As LBG is a non-gelling, linear polysaccharide, it does not form a self-supporting matrix on its own. Without a complementary gelling agent, its ability to hold water was not sufficient to maintain shape stability under thermal conditions.

A1–A4 (A4M + agar combinations):

The A1–A4 formulations, which combined A4M with agar, also showed suboptimal performance. Although agar is a thermoreversible gelling agent that forms hydrogen-bonded networks upon cooling, it did not provide adequate structural support within the carrot purée matrix. This may be attributed to the complex biochemical nature of the purée—rich in pectins, salts, organic acids, and phytochemicals—which likely interfered with agar’s coil-to-helix transition and hindered the formation of stable junction zones. These competitive hydration and ionic interactions [20] may have weakened the gel network, resulting in high syneresis and noticeable structural collapse after steaming.

C1–C4 (A4M + xanthan gum):

The C1–C4 formulations, which combined A4M with xanthan gum, showed reduced syneresis but failed to retain their moulded shape. Although xanthan is known for its high water-binding capacity and ability to increase viscosity—attributed to its stiff helical structure and extensive hydration shell—it does not form a true gel network. Moreover, its interaction with A4M may have been antagonistic. Jo and Yoo [33] reported that thermogelation of cellulose-derived gums like HPMC can be disrupted in mixed hydrocolloid systems due to unfavourable polymer–polymer interactions, which interfere with gel network development and compromise elasticity. A similar mechanism is likely involved: xanthan may have disrupted A4M’s hydrophobic associations by altering local hydration dynamics—namely, by competing for water, modifying water mobility, and interfering with the spatial arrangement of water molecules required for proper gelation. This, along with potential restrictions on polymer chain mobility, likely compromised A4M’s ability to form a cohesive thermogel network, ultimately weakening the overall gel structure.

D1 and D2 (A4M + CMC):

The D1 and D2 formulations, combining A4M with CMC, also failed to maintain structural integrity after steaming. While CMC is effective in increasing viscosity—primarily through electrostatic repulsion and expansion of its polyelectrolyte chains—and is known to enhance moisture retention, it does not form stable gel networks or junction zones. In the D2 sample, which contained a higher concentration of CMC, a modest reduction in syneresis was observed. However, this did not translate into improved shape retention, highlighting a key limitation of viscosity modifiers that lack the ability to contribute to cohesive network formation.

### 3.4. Mechanistic Implications

**Figure 6:**
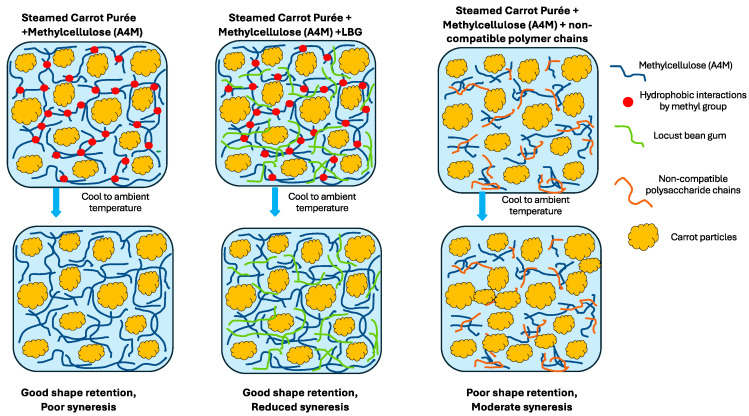
Schematic representation of the proposed mechanisms governing structure formation in steamed carrot purées with different hydrocolloid systems. The figure illustrates the role of methylcellulose (A4M) in promoting thermal gelation via hydrophobic interactions upon cooling, further enhanced by synergistic blending with locust bean gum (LBG), which promotes network entanglement and water immobilisation. In contrast, systems containing non-compatible polysaccharides fail to integrate effectively, resulting in poor shape retention and increased syneresis.

Taken together, the results demonstrate that binary hydrocolloid systems can provide enhanced structural and water-binding functionality in complex vegetable purées. A4M provides thermal gelation, while LBG supports network integrity and water immobilisation via hydrogen bonding and molecular entanglement. Conversely, systems relying solely on viscosity (e.g., xanthan or CMC) or disrupted gelation (e.g., agar) are ineffective in supporting self-standing structures under steaming conditions. These mechanistic distinctions are further illustrated in the microstructures presented in Figure 6, where the methylcellulose (A4M) and A4M–LBG systems exhibit compact, cohesive gel networks with reduced syneresis, in contrast to the disrupted matrices observed in non-compatible gum systems. The findings highlight the importance of matching hydrocolloid functionality—thermal responsiveness, gelation, and molecular interaction—to the physical demands of dysphagia-friendly foods. While this study focused on shear-dominated rheological and structural properties relevant to IDDSI Level 4 requirements, extensional rheology has been increasingly recognised as a crucial parameter influencing bolus cohesiveness and safe swallowing [34]. For instance, Mamaku gum has demonstrated markedly high extensional viscosity due to hydrogen bond-mediated associations, which may support bolus integrity under elongational flow [35,36]. Although not assessed here, such mechanisms may also contribute to the reheated purée’s structural integrity and warrant further investigation.

### 3.5. Validation of Hydrocolloid System in Carrot Purée Using Food Mould

To validate the performance of the optimised hydrocolloid systems under realistic preparation conditions, carrot purées incorporating the selected formulations—B2 (Benecel™ A4M 1.5% + LBG 0.5%) and B3 (Benecel™ A4M 1.5% + LBG 1%)—were shaped using commercially available carrot-shaped silicone moulds (Purée Food Moulds, Australia). The shaped purées were frozen and subsequently steamed, replicating typical food service protocols used in aged-care and hospital settings for dysphagia management.

**Figure 7:**
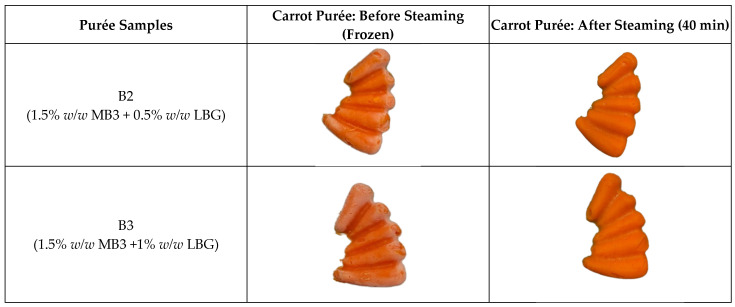
Visual comparison of moulded carrot purée formulations before and after steaming. Carrot purées incorporating hydrocolloid formulations B2 (Benecel™ A4M 1.5% + LBG 0.5%) and B3 (Benecel™ A4M 1.5% + LBG 1%) were shaped using carrot-shaped silicone moulds, frozen, and reheated by steaming. Both formulations maintained structural integrity and recognisability after processing, demonstrating suitability for dysphagia-friendly food service.

Both B2 and B3 systems were compliant with IDDSI Level 4 criteria and demonstrated excellent shape retention and visual integrity after steaming. As shown in Figure 7, these moulded purées preserved their recognisable carrot shape, suggesting effective resistance to thermal deformation. This structural fidelity is particularly relevant for increasing meal recognisability, which has been linked to improved mealtime satisfaction and increased food intake among individuals with dysphagia [15].

Importantly, syneresis was minimal, especially in formulation B3, indicating enhanced water-binding capacity of the composite hydrocolloid matrix—well within the IDDSI stipulation that no thin liquid should leak from Level 4 purées [21]. This reduction in moisture loss contributes not only to the visual appeal and consistency of the product but also to its safety and acceptability—two critical parameters in dysphagia nutrition.

These findings reinforce the potential of B2 and B3 as functionally stable and visually appealing hydrocolloid systems for texture-modified foods. Their compatibility with standard moulding and reheating processes makes them promising candidates for broader adoption in clinical and institutional settings.

## 4. Conclusions

This study demonstrates that methylcellulose (Benecel™ A4M), particularly when combined with locust bean gum (LBG), forms a thermoreversible gel matrix capable of preserving the structural and textural integrity of moulded carrot purée following steaming. Among the systems tested, B2 (A4M 1.5% + LBG 0.5%) and B3 (A4M 1.5% + LBG 1%) exhibited the most favourable outcomes. Both formulations met IDDSI Level 4 criteria, significantly reduced syneresis, and maintained shape fidelity, indicating their suitability for reheatable, texture-modified foods designed for individuals with dysphagia. In contrast, systems containing agar or xanthan gum were less effective, showing poor shape retention and signs of matrix incompatibility. These findings highlight the critical role of synergistic gelation and thermal compatibility in the formulation of dysphagia-friendly foods. Beyond structural benefits, such hydrocolloid systems may also confer physiological advantages, such as modulation of glycaemic response, though this requires further investigation. Additionally, the alignment of instrumental texture measurements with sensory perception—previously shown in related carrot purée studies — further supports the real-world potential of these formulations in clinical and aged-care settings. While this study intentionally focused on instrumental texture and IDDSI compliance, a formal sensory study with the target dysphagic population—assessing flavour, mouthfeel and perceived ease of swallowing—remains a necessary next step and is planned for future work. Future research should expand on these findings by exploring sensory attributes, long-term storage stability, and applicability across a broader range of purée matrices. Future work will also quantify the viscoelastic properties (G′, G″ and sol–gel transitions) of the binary hydrocolloid systems B2 and B3 under heating–cooling cycles to confirm the mechanistic basis for the enhanced height retention and reduced syneresis reported here. While this study primarily focused on shear-dominated rheological behaviour and structural resilience, the role of extensional properties in bolus cohesiveness and swallowing safety remains an important area for further exploration. Collectively, these findings offer a promising framework for the development of reheatable, visually structured purées that combine hydrocolloid functionality with the specific dietary needs of individuals living with dysphagia.

## Figures and Tables

**Table 1:** Optimised formulations of carrot purée.

Sample	Carrot Purée (%)	Potable Water (%)	A4M (%)	Agar (%)	Locust Bean Gum (%)	Xanthan Gum (%)	Carboxy-Methylcellulose (%)
Control	75	25	-	-	-	-	-
A1	75	23.5	-	1.5	-	-	-
A2	75	23.5	0.75	0.75	-	-	-
A3	75	23.5	0.5	1	-	-	-
A4	75	23.5	1	0.5	-	-	-
B1	75	24.5	-	-	0.5	-	-
B2	75	23	1.5	-	0.5	-	-
B3	75	22.5	1.5	-	1	-	-
B4	75	22.5	2	-	0.5	-	-
C1	75	24.5	-	-	-	0.5	-
C2	75	23	1.5	-	-	0.5	-
C3	75	22.5	1.5	-	-	1	-
C4	75	22.5	2	-	-	0.5	-
D1	75	24	-	-	-	-	1
D2	75	22.5	1.5	-	-	-	1

**Table 2:** Sol–gel and gel–sol transition temperatures of methylcellulose and hydroxypropyl methylcellulose solutions, and corresponding G′ values at 90 °C.

Samples	Sol–Gel (°C)	Gel–Sol (°C)	G’ at 90 °C (Pa)
MA (A4C)	62	33	~122
MB (A4M)	57	32	~349
MC (MX)	45	(irreversible)	~208
MD (MX100)	49	(irreversible)	~367
HA (E4M)	63	51	~3

## Data Availability

Data supporting the findings of this study will be made available in a public repository upon publication (https://masseyuni-my.sharepoint.com/:f:/g/personal/ktgoh_massey_ac_nz/Em-jqAYi7shLiSb6iDX_F9UBSjeyle941zypXyUjthhasQ?e=gqYHSk, accessed on 22 June 2025).

## Abbreviations

The following abbreviations are used in this manuscript:

ANOVA	Analysis of Variance
CMC	Carboxymethylcellulose
G′	Storage Modulus
G″	Loss Modulus
HPMC	Hydroxypropyl Methylcellulose
IDDSI	International Dysphagia Diet Standardisation Initiative
LBG	Locust Bean Gum
LVR	Linear Viscoelastic Region
MC	Methylcellulose
SD	Standard Deviation
